# Study on Microstructure and Mechanical Properties of Laser Welded Dissimilar Joint of P91 Steel and INCOLOY 800HT Nickel Alloy

**DOI:** 10.3390/ma14195876

**Published:** 2021-10-07

**Authors:** Vishwa Bhanu, Dariusz Fydrych, Ankur Gupta, Chandan Pandey

**Affiliations:** 1 Department of Mechanical Engineering, Indian Institute of Technology Jodhpur N.H. 62, Nagaur Road, Karwar, Jodhpur 342037, India; bhanu.1@iitj.ac.in (V.B.); ankurgupta@iitj.ac.in (A.G.); 2 Faculty of Mechanical Engineering and Ship Technology, Institute of Machines and Materials Technology, Gdańsk University of Technology, Gabriela Narutowicza Street 11/12, 80-233 Gdańsk, Poland

**Keywords:** P91, Incoloy 800HT, laser welding, weld fusion zone, weld fusion boundary, heat-affected zone, post-weld heat treatment, solidification cracking, tensile strength, impact toughness

## Abstract

This investigation attempts to explore the weld characteristics of a laser welded dissimilar joint of ferritic/martensitic 9Cr-1Mo-V-Nb (P91) steel and Incoloy 800HT austenitic nickel alloy. This dissimilar joint is essential in power generating nuclear and thermal plants operating at 600–650 °C. In such critical operating conditions, it is essential for a dissimilar joint to preserve its characteristics and be free from any kind of defect. The difference between the physical properties of P91 and Incoloy 800HT makes their weldability challenging. Thus, the need for detailed characterization of this dissimilar weld arises. The present work intends to explore the usage of an unconventional welding process (i.e., laser beam welding) and its effect on the joint’s characteristics. The single-pass laser welding technique was employed to obtain maximum penetration through the keyhole mode. The welded joint morphology and mechanical properties were studied in as-welded (AW) and post-weld heat treatment (PWHT) conditions. The macro-optical examination shows the complete penetrations with no inclusion and porosities in the weld. The microstructural study was done in order to observe the precipitation and segregation of elements in dendritic and interface regions. Solidification cracks were observed in the weld fusion zone, confirming the susceptibility of Incoloy 800HT to such cracks due to a mismatch between the melting point and thermal conductivity of the base metals. Failure from base metal was observed in tensile test results of standard AW specimen with a yield stress of 265 MPa, and after PWHT, the value increased to 297 MPa. The peak hardness of 391 HV was observed in the P91 coarse grain heat-affected zone (CGHAZ), and PWHT confirmed the reduction in hardness. The impact toughness results that were obtained were inadequate, as the maximum value of impact toughness was obtained for AW P91 heat-affected zone (HAZ) 108 J and the minimum for PWHT Incoloy 800HT HAZ 45 J. Thus, difficulty in obtaining a dissimilar joint with Incoloy 800HT using the laser beam welding technique was observed due to its susceptibility to solidification cracking.

## 1. Introduction

The modern era is set to explore more efficient and eco-friendly sources of power generation. A prime focus is to improve the working efficiency of current nuclear and thermal power plants by increasing their operating temperatures. High operating temperatures reportedly reduce carbon emissions [1]. The most frequently employed material in a nuclear power plant is P91 (ASTM A335) [2], because of its creep-enhanced properties and low cost. It is used for constructing the majority of parts operating at 600–620 °C, such as header sections that are connected to tubes carrying high-temperature steams [3,4,5]. However, the poor oxidation resistance of P91 steel limits the life of the component up to a working temperature of 610 °C [6]. The tubes operating at around 650–700 °C are generally made up of higher heat resistance and oxidation corrosion-resistant super-advanced alloys; Incoloy 800HT is one of these. In a weldment, the Incoloy 800HT base material is the liming factor which is operatable up to only 700 °C, while its heat-affected zone (HAZ) and weld can operate at and above 800 °C [7]. Hence, studying this combination of the dissimilar joint is necessary. Recently Gas Tungsten Arc Welding (GTAW) has been used to make such dissimilar welds that require edge preparation and involve high thermal stress induced due to multiple weld passes [8,9]. While GTAW is not a suitable process for obtaining higher penetration in a single pass, laser welding comes to the rescue [10]. GTAW also requires careful selection of filler material which could provide the transition in coefficient of thermal expansion (CTE) between the two dissimilar metals. As dissimilar joints face the issue of difference in thermal conductivity, melting point, and carbon migration, the employment of recommended Ni-based alloys can effectively act as perfect transitional alloys to alleviate such problems [11]. Due to the presence of slightly high concentrations of Mo, V, Nb, and Ni, P91 has a martensitic structure and solid solution strengthening mechanism which gives the material desirable strength under high temperatures. Precipitate formation in P91, such as M_23_C_6_ and MX, imparts precipitation hardening [12]. The austenitic Incoloy 800HT (ASTM B409) has a comparatively larger grain size which gives it enhanced creep strength and ductility [13]. The Ni-Fe-Cr system of Incoloys is generally stable in the austenitic phase, and the solidification of the weld is also completely austenitic in nature. In addition, carbides, nitrides, and phases like TiC, TiN, M_23_C_6_, γ’(Ni_3_(Al, Ti)), and γ’’(Ni_3_Nb), are prime strengthening phases [14,15]. Elements like Co, Cr, Fe, Mo, W, and Ta in Incoloy 800HT provide solid solution strengthening [16]. Incoloy 800HT is compositionally different from its parent material, as carbon (C) and Al+Ti percentages are slightly more precisely controlled. Both materials are prone to the formation of detrimental Laves and σ phases under high temperatures during their service life [17]. Incoloy 800HT is reportedly prone to solidification cracking caused by uncontrolled heating and cooling, exposure to sulphur, and segregation along dendrites [18,19]. The laser-welded joining of martensitic and austenitic alloys was reported with pores, inclusion, and delta ferrite formation [20]. Works from other authors also suggest controlling the formation of delta ferrite in the weld in order to reduce the solidification cracking [21]. Laser beam welding has proved successful for obtaining the dissimilar weldments that are required to operate in critical conditions in a powerplant [22]. The concern of the untempered martensite formation in the P91 HAZ causes the reduction in impact toughness, which, reportedly, can be enhanced by PWHT. In addition, it is well documented that post-weld heat treatment (PWHT) at an optimum temperature of 760 °C effectively reduces the magnitude of hardness across the weld, and aging does not impart any effect on the mechanical properties of the weld [23,24].

Various works have been reported related to the dissimilar welded joint of P91 with austenitic alloys, such SS 304 [25], SS 316LN [26], SS 304L [27,28], and with nickel alloy interlayers of Incoloy 800H [11,29]. In these studies, the selection of nickel-based alloys is found to be beneficial, as they may provide a transition in terms of the coefficient of thermal expansion and have reportedly controlled carbon migration into the austenitic side. However, the alloying elements present in the nickel-based alloys tend to provide a lower rate of diffusion. The lower diffusion rate in the matrix contributes to a higher solidification rate, leading to heavy micro-segregations while solidification. The martensitic side also witnesses the formation of unwanted soft delta ferrite patches and variation in microhardness near the weld fusion boundary [30,31]. Studies have shown a reduction in the mechanical properties of Incoloy 800HT welds due to the high heat input during the GTAW process. As per reports, nickel has a high affinity for sulphur, which attacks the grain boundaries forming harmful nickel sulphide and making it prone to solidification cracking [32]. The dissimilar welding of the Cr-Mo ferritic steel and austenitic Incoloy 800 was observed with a softening of the intercritical heat-affected zone, leading to Type IV failure and susceptibility to creep cavitation due to the formation of coarse carbide particles in the weld fusion zone near the weld fusion boundary [33]. In a notable study, a successful weldment of modified 9Cr-1Mo steel and alloy 800 was obtained using Inconel 82 filler and this confirmed the reduction in carbon migration and showed the negligible effect of the ageing process on impact toughness [34]. The employment of activating flux in GTAW, better known for increasing the depth of penetration through the reversal of Marangoni flow in the weld pool, was reported for welding Incoloy 800H. In the reported study, the usage of MoO_3_ flux resulted in solidification cracking, though an increase in impact toughness was observed with the usage of TiO_2_ flux [35].

The unconventional CO_2_-based laser welding technique is still unexplored in terms of making a dissimilar weld of P91 and Incoloy 800HT. The lack of discussion in the very few reported works on microstructural and mechanical properties regarding the joining of P91-Incoloy 800HT inspires further investigation into the dissimilar welding of P91 with the successor Incoloy 800HT in terms of powerplant based applications.

## 2. Materials and Methods

The P91 steel plate was received in normalized and tempered condition (normalizing: 1040 °C/10 min, followed by air cooling; tempering: 760 °C/120 min, followed by air cooling) while the as-received Incoloy 800HT plate was in a solution-annealed condition, as per the manufacturer. The chemical composition of the material was determined using the optical emission spectrometer (OES) (Metalvision-1008i, Metal Power Analytical Pvt. Ltd., Mumbai, India) and is presented in Table 1. To obtain the desired weld penetration for the experiment, plates with dimensions of 120 mm × 50 mm × 6 mm were extracted from the as-received material. The plates were ground on the faces using a surface grinder in order to make an intimate contact of faying surfaces. Additionally, the plates were cleaned using acetone before welding to exclude any foreign particles, grease, dust, and rust. The dissimilar laser welded joint was made in a flat (1G) position using a CO_2_-based laser (Laser line LDF4000-30 Diode Laser, Laserline GmbH, Hamburg, Germany), and the operating parameters are given in Table 2.

After welding, the plate was allowed to cool down at room temperature. The two sets of the welded joint were prepared. One set of the welded plate was employed for testing in the as-welded (AW) condition, and the other set was subjected to post-weld heat treatment (PWHT). The welded plate was allowed to cool in air that was up to a temperature of 100 °C in order to minimize the effect of the diffusible hydrogen content, and PWHT was then carried out at a temperature of 760 °C for 2 h [36].

**Figure 1:**
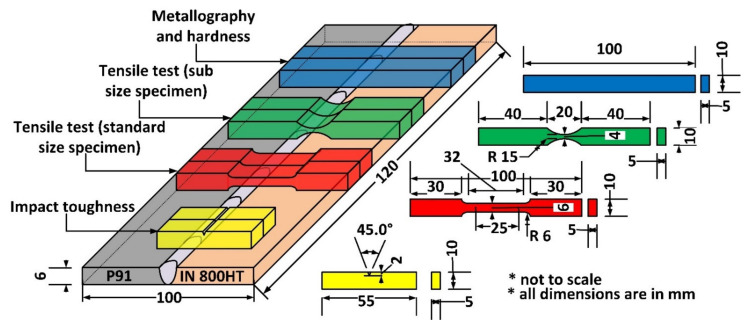
Schematic representing the extracted specimen’s dimensions.

The PWHT was performed in an electric furnace and was air-cooled, as it is necessary to remove the untempered martensite formed in the HAZ region [37]. During the visual tests, it was found that a clean, perfect weld bead was obtained. The metallographic and material characterization samples were extracted from the welded plate as per the dimensions depicted in Figure 1. The test specimens were extracted using a high-speed Bandsaw machine as per the required dimensions. The extracted specimens for metallographic observation were mirror-polished using different grades of emery paper, polishing cloth, and diamond paste on an 8-inch disk size polishing machine. For microstructural characterization, the polished samples were etched. Villella’s reagent (1 g picric acid, 5 mL hydrochloric acid, and 100 mL ethanol) was used for etching P91 steel. The specimen was immersed in Villella’s reagent for 60 s. The electrolytic etching (100 g oxalic acid solution in 100 mL distilled water) at 6 V was done for weld fusion zone (WFZ), Incoloy 800HT base metal, and HAZ. Under electrolytic etching, the specimen was etched for 60 s. Macro optical observations were made on a stereo microscope, while micro-optical analysis of the microstructure was completed with an optical microscope (Make: Leica, Model: DMC4500, Leica Microsystems GmbH, Wetzlar, Germany).

For a detailed analysis of different phases and precipitates, a scanning electron microscope (SEM) equipped with energy dispersive spectroscopy (EDS) (Carls Zeiss Ultra plus and FEI Quanta 200, Carl Zeiss Microscopy Deutschland GmbH, Oberkochen, Germany) was employed. Microhardness tests were done at 0.5 kgf load with a dwell time of 10 s on an automatic Vickers microhardness tester (Mitutoyo, Model: Autovick HM-200, Mitutoyo, Kawasaki, Japan). Tensile tests were performed as per ASTM 370 [38] standards at a 1 mm/min strain rate on a tensile testing machine (Instron 5980 of 100 kN capacity, Instron, Massachusetts, USA). The Charpy impact toughness of the WFZ and HAZ was evaluated for a standard impact toughness specimen with dimensions of 55 mm × 10 mm × 5 mm and a central V notch of depth 2 mm. The Charpy V impact test for WFZ and HAZ was conducted using an Impact tester (FIT-400-ASTM-D, Fine Testing Machines Pvt. Ltd., Miraj, India) with a capacity of 400 Joule.

## 3. Results and Discussion

### 3.1. As-Received Material Microstructure

**Figure 2:**
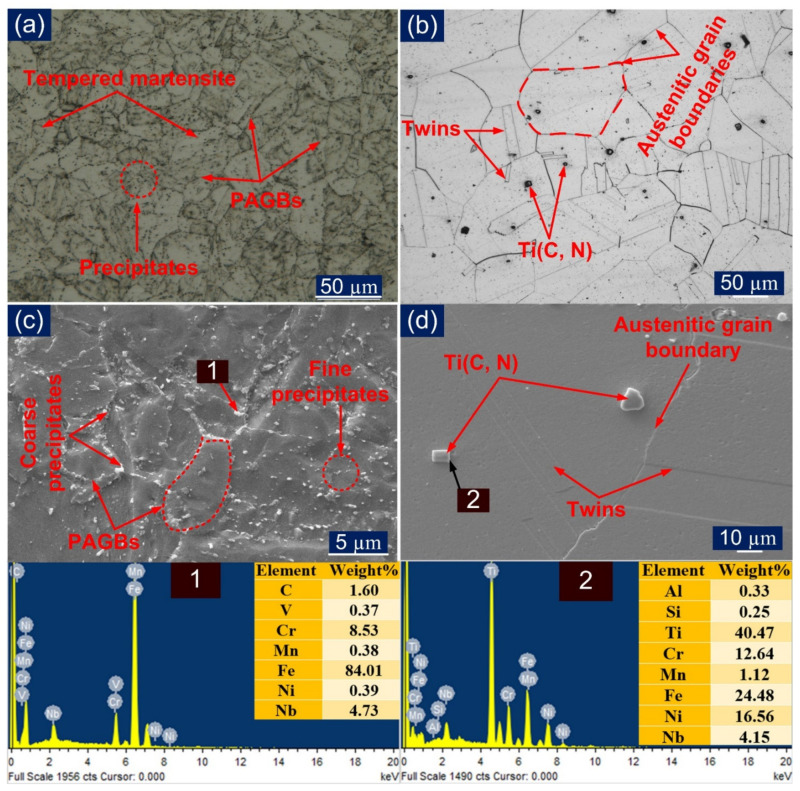
(**a**) P91 base metal optical microstructure; (**b**) Incoloy 800HT base metal optical microstructure; (**c**) P91 base metal microstructure under SEM with EDS spectra of the precipitate; (**d**) Incoloy 800HT base metal microstructure under SEM with EDS spectra of the precipitate.

The microstructure of the as-received material was observed under an optical microscope and SEM. The microstructure of P91 is shown in Figure 2a,c. This confirms the presence of the martensitic structure, which was obtained during the normalizing and distribution of precipitates that evolved while tempering. The precipitation hardening and solid solution hardening give stability to the microstructure at an operating temperature above 500 °C [30]. The presence of prior austenitic grain boundaries (PAGBs) and intra-lath region can be visualized, and higher precipitation along the PAGBs can also be observed. The group of parallel laths forming packets and in each packet locks of martensite laths in the same orientation were witnessed. The reported precipitates for P91 are predominantly M_23_C_6_ (M: Fe, Mo, Mn, Cr) and MX (M: Nb, V; X: C, N) [39,40].

For Incoloy 800HT, the grain structure is presented in Figure 2b,d and was found to consist of a homogeneous and austenitic matrix phase. Somewhat larger grains were observed with an austenitic structure and abundant annealed twin formations [17]. The mechanical properties of both P91 and Incoloy 800HT are presented in Table 3. The large grain size in Incoloy 800HT is attributed to the solution annealed condition, which imparts a high strength, creep resistance, and high resistance to rupture at elevated operating temperatures.

Different precipitate particles were spotted in the matrix. The matrix was free from any detriment impurities, delta-ferrites, and sigma phase. The EDX analysis of precipitates found in the base Incoloy 800HT is shown in Figure 2d. The average grain size for Incoloy 800HT was found out to be 75.7 ± 10 μm and the average size of PAGs of P91 was 13 ± 5 μm.

### 3.2. Characterization of the Weldments in As-Welded (AW) and Post-Weld Heat Treatment (PWHT) Condition

**Figure 3:**
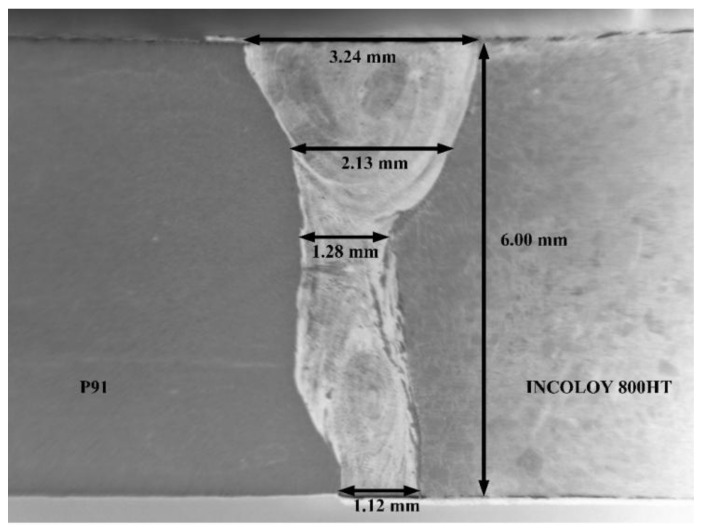
Macrograph of the laser weld bead.

The macrograph of the weld cross-section representing the weld bead geometry in Figure 3 hints at the keyhole mode of penetration caused by vapor pressure under high heat energy input in laser welding. Complete penetration with a narrow weld bead was obtained, as per the macrograph. Due to heat transfer and conduction, a grain size gradient was formed along the weldments. The weldment obtained and its different zones can be further characterized in detail.

#### 3.2.1. Heat-Affected Zones

**Figure 4:**
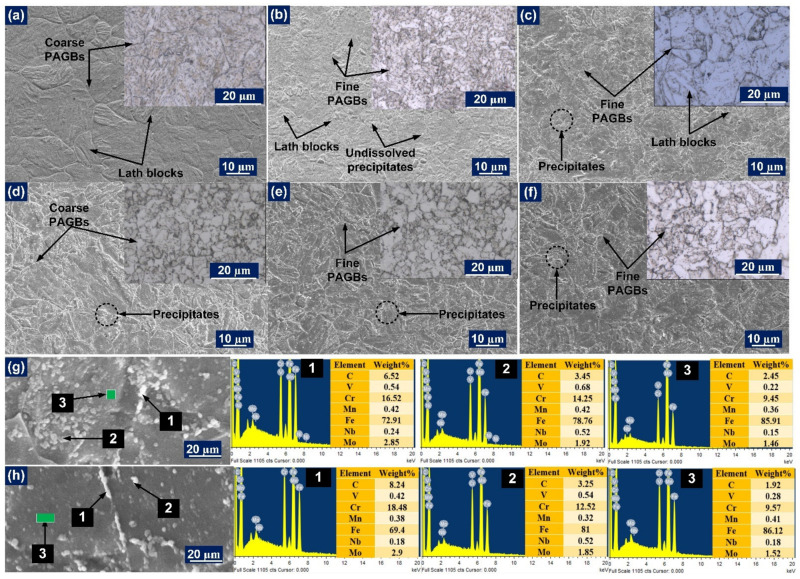
P91 HAZs in AW condition: (**a**) CGHAZ; (**b**) FGHAZ; (**c**) ICHAZ; after PWHT (**d**) CGHAZ; (**e**) FGHAZ; (**f**) ICHAZ; ICHAZ at higher magnification showing the location for EDS spectrum for (**g**) AW; (**h**) PWHT condition.

The micrograph of the P91 steel HAZ next to the WFZ consists of three different regions with an inhomogeneous microstructure. On the basis of the peak temperature experienced and phase transformations during the weld thermal cycle, the P91 HAZ typically consists of the coarse-grained HAZ (CGHAZ), fine-grained HAZ (FGHAZ), and inter-critical HAZ (ICHAZ) [41]. The microstructure of the CGHAZ adjacent to the WFZ experiences a much higher peak temperature than the A_c3,_ and, due to exposure at such a high temperature, precipitates are dissolved, which contributes to the reduction of the pinning force at the grain boundaries and results in grain coarsening, as shown in Figure 4a. The optical image Figure 4a shows the presence of the lath blocks and coarse grains in CGHAZ, which were also observed in the SE image. The SE image of CGHAZ also shows the complete dissolution of the particles. The region of FGHAZ and ICHAZ both have fine-grain structures and were difficult to distinguish by optical image, as shown in Figure 4b–d. However, the peak temperature experienced by the FGHAZ was higher or close to A_c3,_ and, for ICHAZ, it was in the range of A_c1_ to A_c3_. That difference in peak temperature experienced by the FGHAZ/ICHAZ leads to the variation in the overall fraction area of the precipitates and final microstructure. FGHAZ exhibits the untempered martensitic microstructure and partial dissolution of the carbide precipitates due to the lower peak temperature and results in the formation of the fine PAGs compared to CGHAZ Figure 4b. The undissolved carbide precipitates in FGHAZ show the coarsening nature and are marked in Figure 4b. In ICHAZ, the lower peak temperature results in the partial transformation of the austenite and the negligible dissolution of the carbide precipitates. This results in the final ICHAZ microstructure which exhibits the complex structure of the untempered martensite and austenite transform products (ATP), as given in Figure 4c. Although all of the regions of P91 HAZ of the weldments differ significantly in their PAGs, the fraction area of the precipitates, and the final microstructure, after PWHT the phase of each zone was basically the same and consisted of the ferritic-carbide mixture, i.e., tempered martensite and carbide precipitates with different tempering degree depending on the peak temperature experienced during the welding cycle. The region of the HAZs of P91 steel after the PWHT is mentioned in Figure 4d–f. After PWHT, however, more precipitates evolved at the grain boundaries and matrix. This caused softening across the HAZ due to a reduction in solid solution hardening [42]. The CGHAZ, after PWHT, exhibit the coarse PAGs in the tempered martensitic matrix (Figure 4d: optical image) along with newly evolved carbide and carbonitrides precipitates along the boundaries and inside the matrix (Figure 4d). The evolution of the new precipitates was also observed in FGHAZ and ICHAZ, as given in Figure 4e,f. The higher magnification image was captured in the ICHAZ for both AW (Figure 4g) and PWHT (Figure 4h) conditions to observe the decoration of the particles along the boundaries and inside the matrix. The image also shows the morphology and distribution of the precipitates inside the matrix and is also utilized for the EDS spectrum. The EDS spectrum of the white particles (Figure 4g: AW) decorated along the PAGBs shows the higher weight percentage of the Cr (16.52%) and Mo (2.85%) which ensure the presence of the secondary phase carbide particles that are enriched with Cr and Mo. The white particles inside the matrix show chromium (Cr) and molybdenum (Mo) as major elements, along with vanadium (V) and niobium (Nb). The EDS spectrum of the matrix is represented at point 3. A similar observation was also observed after the PWHT and is represented in Figure 4h. It is also well known from other studies that P91 steel contains mainly intragranular MX precipitates enriched with V and Nb, and intergranular M_23_C_6_ precipitates at PAGBs and sub-grain boundaries mainly enriched with Cr and Mo.

**Figure 5:**
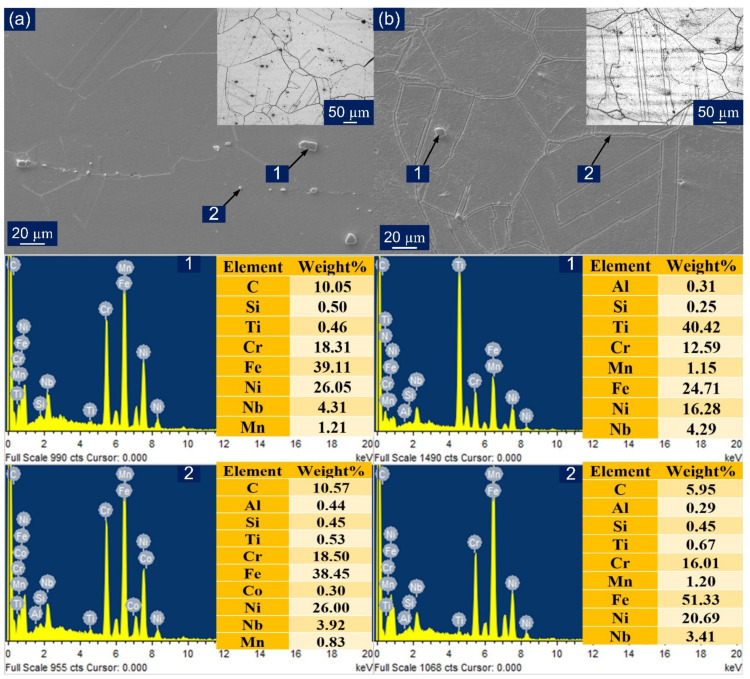
(**a**) Incoloy 800HT HAZ in AW condition with EDS spectrum for precipitate observed in AW condition; (**b**) Incoloy 800HT HAZ in PWHT condition with EDS spectrum for precipitate observed in PWHT condition.

While a coarse grain can be observed near the weld fusion boundary of Incoloy 800HT, a very narrow and indistinguishable HAZ was formed owing to its comparatively lower heat conductivity. The microstructure of Incoloy 800HT was found to be constant and homogenous in the welded state. The grains that are near to the weld fusion boundary of Incoloy 800HT are classified as CGHAZ (Figure 5a,b: optical). The presence of Ti(C,N) is visibly peaked in Figure 5a. The EDS spectra result for the thickened grain boundary with the possibility of grain boundary liquation is shown in Figure 5b via spectrum at point 2; a high Cr percentage can be inferred to the formation of M_23_C_6_ precipitate near grain boundaries. However, the PWHT performed for this study has not shown any effect on the Incoloy 800HT base metal and weld fusion boundary, as the recommended PWHT temperature for this is 820 °C, which is higher than the recommended and performed PWHT temperature for P91 weldments [43]. The presence of Cr, Fe, Al, Ti, and Nb in the alloying composition of Incoloy 800HT contributes to solid solution hardening. In addition, the controlled presence of the γ’ phase along with the existence of different carbide phases contributes to precipitation hardening.

#### 3.2.2. Weld Fusion Boundary

**Figure 6:**
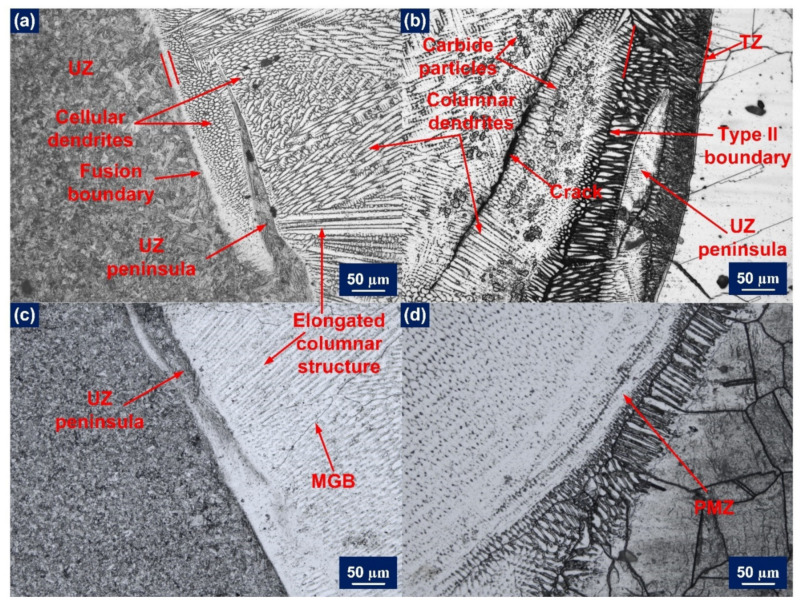
Optical image showing weld fusion boundary of (**a**) AW P91 side; (**b**) AW Incoloy 800HT side; (**c**) PWHT P91 side; (**d**) PWHT Incoloy 800HT side.

The solute segregation phenomenon can be observed at the macroscopic level near the fusion boundary during constitutional supercooling. There is a noticeable inhomogeneity present in chemical composition distribution and microstructure near the weld metal fusion boundary. Every autogenous weld is known to exhibit epitaxial growth, but the nature of a dissimilar weld between austenitic (FCC) metal and martensitic (BCC) metal leads to the formation of a Type II boundary parallel to a fusion boundary that reduces the possibility of epitaxial growth [44]. Figure 6a gives a clear illustration of the AW P91 weld interface in which the fusion boundary, beach or unmixed zone formation, and growth of cells and dendrites can be clearly acknowledged. The presence of macrosegregation can also be established in the form of a peninsula, islands, and beaches. In Figure 6b, the AW Incoloy 800HT weld fusion boundary is illustrated. The illustrated weld fusion boundary has a comparatively thicker transition zone (TZ) after the fusion line [45]. It also has a columnar structure, peninsula, and unmixed or partially mixed zone (PMZ) formation. The difference in the melting temperature of the employed base metals causes the formation of UZ or PMZ. The Incoloy 800HT HAZ has shown thickening of the grain boundaries very adjacent to the fusion line, leading to precipitation of the Ti enriched particles [46]. Similar results were confirmed in the PWHT state. As presented in Figure 6c, the PWHT P91 weld fusion boundary has the unmixed zone and UZ peninsula formation. And in Figure 6d, PWHT Incoloy 800HT weld fusion boundary has explicit PMZ formation and HAZ grain boundary thickening. PWHT does not have any effect on the weld fusion boundary at both sides but only tempers the P91 HAZ martensite.

**Figure 7:**
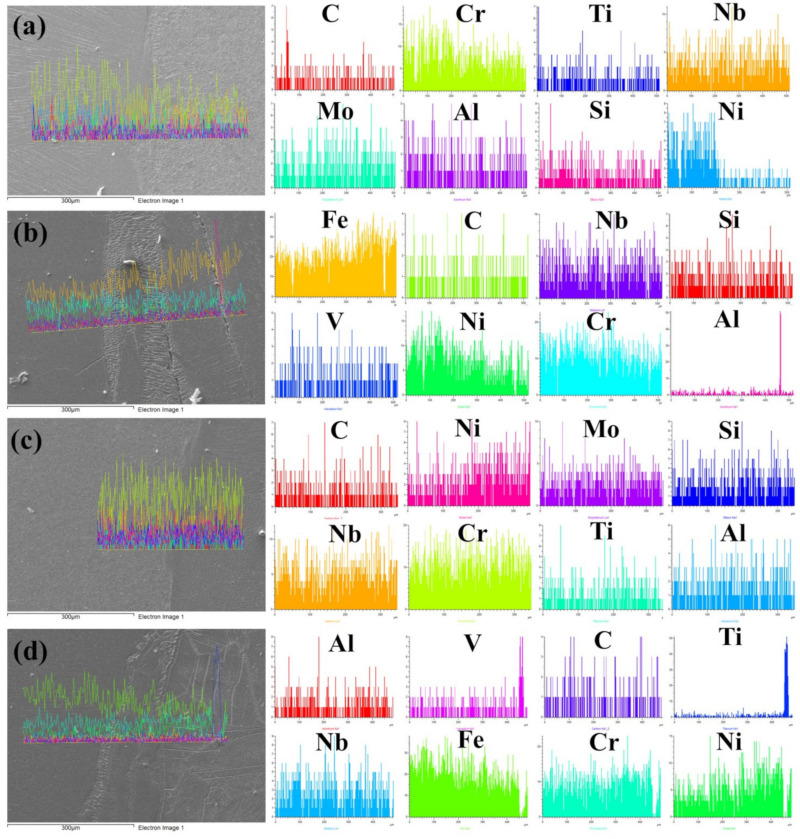
Line map EDS analyses along (**a**) AW P91 weld fusion boundary; (**b**) AW Incoloy 800HT weld fusion boundary; (**c**) PWHT P91 weld fusion boundary; and (**d**) PWHT Incoloy 800HT weld fusion boundary.

As weld fusion boundaries are crucial sites for solute segregation to occur, further analysis of elemental distribution across them was done through EDS linear spectra. Figure 7a has shown a decrease in nickel and chromium composition from weld to base metal P91 in the AW condition, which corresponds to the P91 composition. A slightly increased Mo concentration in the unmixed zone near the fusion boundary was observed. A sudden carbon peak suggests the presence of carbide particle precipitate in the weld zone. If this is noticed to be corresponding to a carbon peak, there is Si peak emerging which could be indicative of M_6_C formation. In the Incoloy 800HT interface, as shown in Figure 7b, expected peaks were obtained for Ni, Cr, and Fe, as per the metal composition. A sudden increased appearance of Al could confirm the presence of the γ’ phase. Interestingly, the PWHT weld fusion boundary of P91, shown in Figure 7c, does not show any significant change in compositional behavior, but a constant distribution of chromium across the weld fusion boundary was witnessed. In the PWHT weld fusion boundary of Incoloy 800HT (Figure 7d), all elements confirm the discussed behavior, and no particular change corresponding to the PWHT was observed. However, in Incoloy 800HT HAZ, a rise in V and Ti can be seen at the spotted precipitate, but no change or peak in carbon concentration was present corresponding to a Ti rise. This confirms the presence of the TiN particles.

#### 3.2.3. Weld Fusion Zone (WFZ)

**Figure 8:**
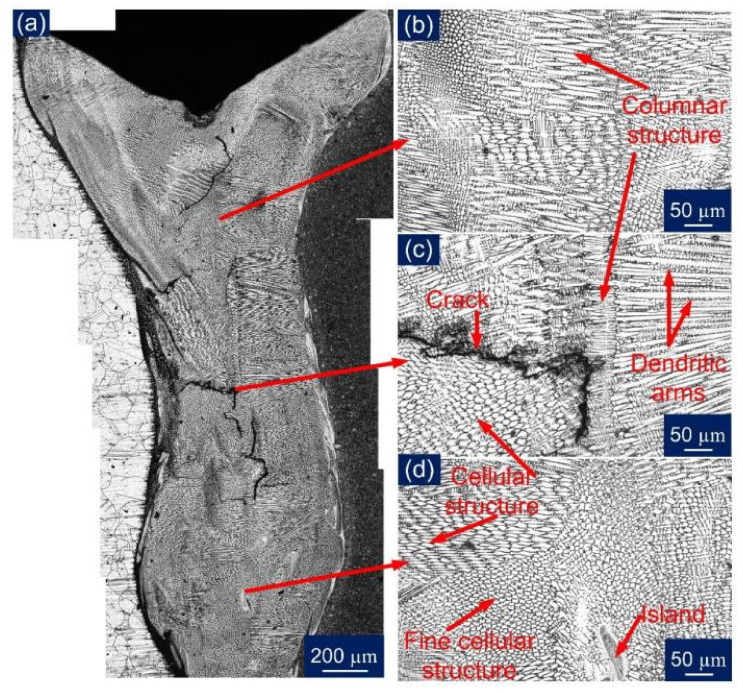
Optical images for: (**a**) WFZ in AW condition; (**b**) top WFZ; (**c**) WFZ mixed zone; (**d**) bottom WFZ.

The optical images were captured for the entire WFZ for detailed analysis, as shown in Figure 8. The different austenitic structures obtained in the austenitic matrix of the WFZ can be categorized on the basis of the solidification mode. The solidified microstructure in the WFZ is controlled by the growth rate (R), temperature gradient (G), degree of the constructional supercooling, and alloy composition. The ratio G/R determines the solidification mode, and G*R influences the size of the structure. The growth occurs from unmelted solid grains of the base metal; hence the G/R ratio is higher at the fusion boundary. The degree of constitutional supercooling affects the temperature gradient, transforming the solidification mode to form cellular, columnar, and equiaxed dendrites. The solidification tends to proceed from the interface to the WFZ. Similarly, different dendritic structures were obtained in the WFZ. It can be perceived, from Figure 8a, that in the formation of the different structures throughout the WFZ, the columnar growth predominated near the base metal interface, and the solidification mode then changed to the cellular and fine cellular structure in the WFZ. Figure 8b depicts a mixed zone in the WFZ where different dendritic morphologies exist. The dendritic arms and inter-dendritic cores can also be observed where segregation of alloying elements occurs. Figure 8c illustrates the solidification cracking phenomenon observed along the solidified grain boundary (SGB). The dominant presence of different cellular structures and an unmixed zone ‘island’ (Figure 8d) were also spotted near the bottom of the WFZ. The different cells or dendrites were separated by a boundary that has a different composition from the bulk weld fusion zone, due to solute redistribution known as the solidification subgrain boundary (SSGB). The intersection boundary of such a similar group of subgrains or cells can be described as a solidification grain boundary. At the same time, a high-angle misorientation SGB whose crystallographic component migrates away from its compositional component can be described as a migrated grain boundary (MGB).

**Figure 9:**
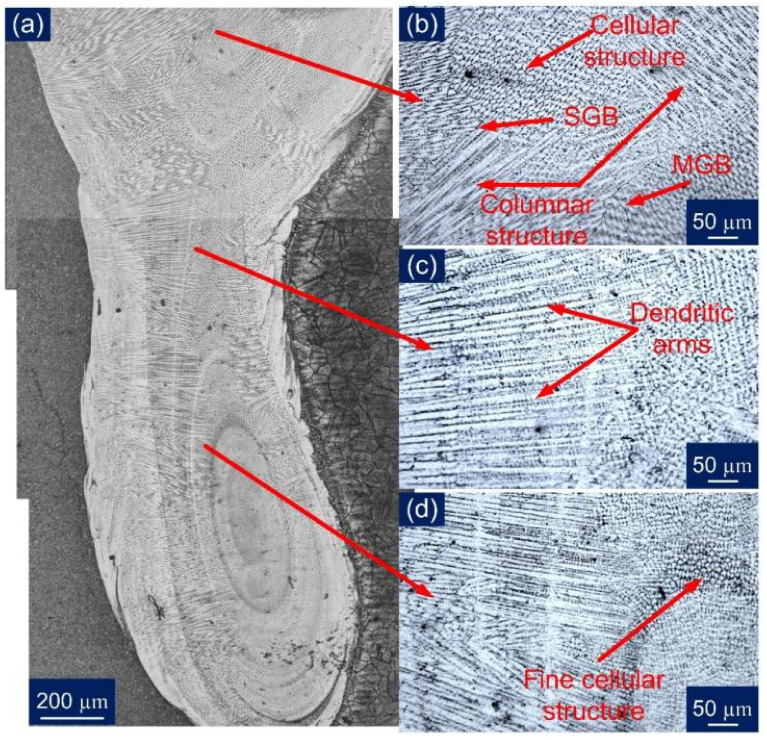
Optical images for: (**a**) WFZ in PWHT condition; (**b**) top of the WFZ; (**c**) WFZ mixed zone; (**d**) bottom WFZ.

Further, the comparative analysis was completed for a PWHT WFZ. However, being austenitic in nature, it did not respond to PWHT. From a stitched Figure 9a, though, it can be assumed that cracks were not present along the overall weld length, as no sign of cracks were there in the few specimens observed. Figure 9b–d also confirms the presence of discussed solidification modes as in the observed top WFZ groups of cellular and columnar structures are indicated with SGB and MGB. It was concluded, from the optical image study, that inhomogeneity was present in the WFZ to a significant extent; hence the inter-dendritic areas were further characterized using EDS analysis.

**Figure 10:**
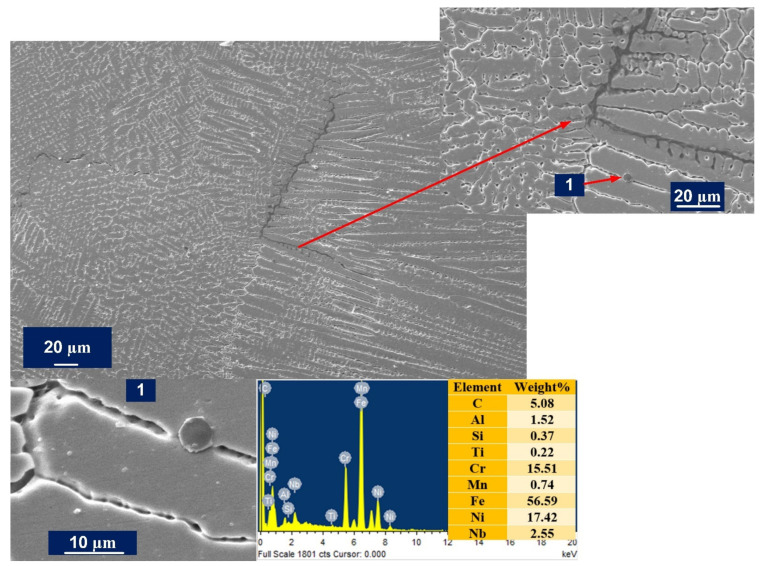
SEM micrograph cracks in AW specimen WFZ and EDS spectra of precipitate observed in the dendritic core region.

**Figure 11:**
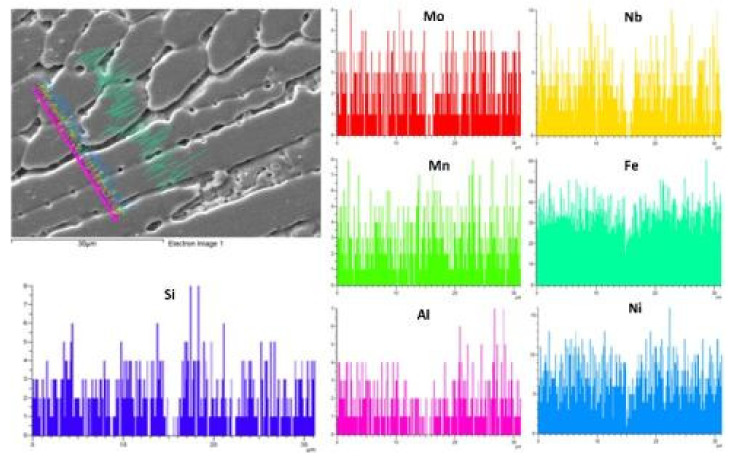
Line map EDS analysis across the interdendritic cores in the weld region.

The solidification cracks were observed in the WFZ along the grain boundaries, as shown in Figure 10, and the EDS analysis suggests the particle might be a chromium carbide. However, Figure 9a indicates no cracks in the WFZ. This could suggest the cracks were not uniformly distributed along the welded joint. In Figure 8a, the crater at the very top indicates that, under the sudden shock of the high temperature at the start, the initial location under the focus of the laser beam—the area to be joined—might have experienced cracking, possibly in a limited area only. The major cause for this could be the presence of low melting eutectic constituents forming elements, i.e., Nb, Ti, and Si. However, no liquation cracking was observed in the HAZ, due to the fine and coarse microstructural differences of the base metals. During solidification, the segregation of Nb occurred in the dendritic areas, as shown in Figure 11. As the eutectic reaction occurs, the formation of γ/NbC and γ/Laves constituents is reported to take place and this expands the solidification temperature range, leading to uneven cooling and cracking [47]. Nickel has a high affinity for sulfur to form nickel sulfide (NiS) along the grain boundaries and is considered to be the main cause of hot cracking in Incoloy 800HT during welding [17]. Type 1A cracks were observed in the weld region only, and no crack formation was witnessed in the HAZ regions of either parent metal. More cracks were observed in the weld region near to Incoloy 800HT side interface, which again reflects the susceptibility of Incoloy 800HT to hot cracking.

In Figure 11, the microsegregation of Mo, Si, and Fe along dendritic core and subgrain boundaries in the interdendritic region can be observed. Meanwhile, the P91 side of WFZ majorly evolved elongated cellular and columnar dendrites. The fusion structures were formed as per the solidification rate, which is affected by the material’s physical and chemical properties and, also, the keyhole mode. Rapid heating and cooling causes the cellular structure to change into a dendritic structure. The keyhole causes a low transfer of heat through the interface to the base metal, which also creates thermally generated stresses. In the weld region, grain boundaries were found to be rich in Ti, Si, and Nb concentrations. These elements tend to produce complex solidification temperature changes at the grain boundaries. Si concentration which was segregated, as shown in Figure 11, was found in higher concentrations in fusion zone, and this plays a crucial role in hot cracking. As the columnar dendritic structure was observed in the WFZ in a large quantity, it is possible that the cracks were observed alongside them, as depicted in Figure 10. Carbon affects the formation of a higher columnar dendritic structure, which causes partial dissolution of precipitates. Fewer precipitates were observed in the WFZ as Ti consumed C present and its carbide transferred in the molten pool and accumulated near the Type II boundary.

### 3.3. Mechanical Properties of the Welded Joint

#### 3.3.1. Tensile Properties

**Figure 12:**
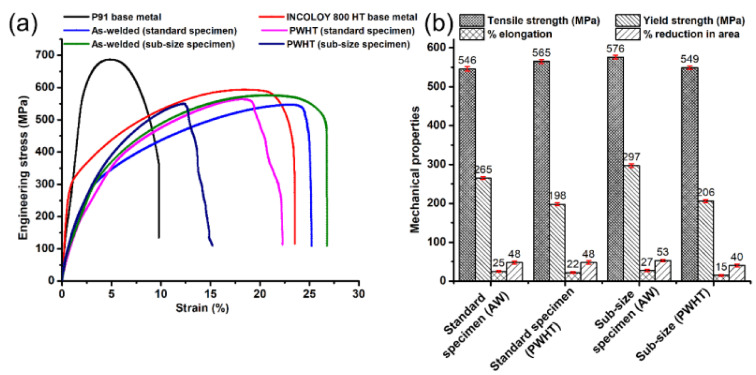
(**a**) Engineering stress-strain curves for performed tensile tests, (**b**) variation in mechanical properties for different conditions of the welded joint.

**Figure 13:**
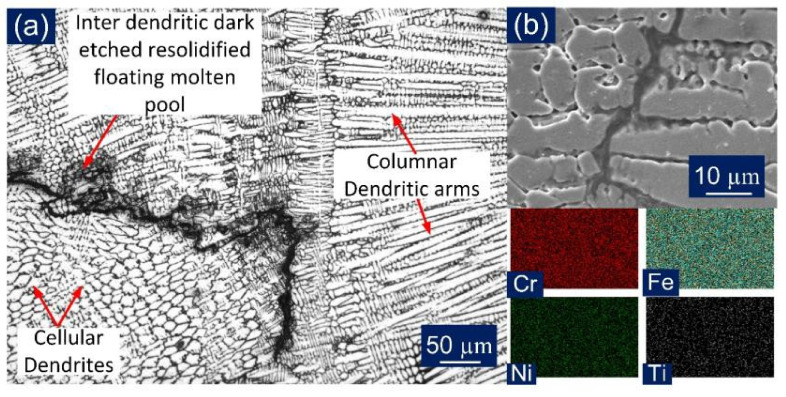
(**a**)The incomplete healing process in the surrounding of the crack in the AW WFZ; (**b**) EDS area mapping data.

**Figure 14:**
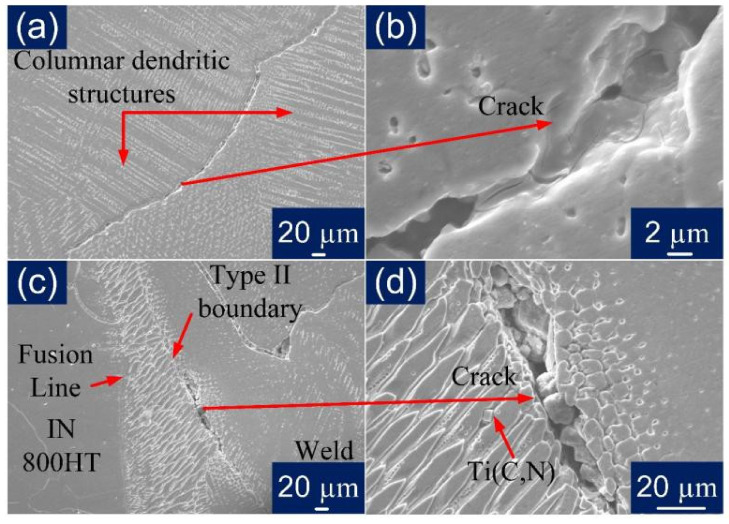
SEM micrograph for PWHT specimen (**a**) WFZ, (**b**) crack along SGB, (**c**) PWHT Incoloy 800HT fusion boundary, (**d**) crack along Type II fusion boundary.

The tensile tests were conducted on the transverse tensile specimen, and the test results are mentioned in Table 4. The trend of stress and strain can be observed and compared through the engineering stress-strain obtained, as shown in Figure 12a. The variation in tensile properties is shown in Figure 12b. The tensile strength of the AW joint (standard specimen) was measured 546 ± 6 MPa, which was lower than the tensile strength of both the base metal P91 (687 ± 8 MPa) and Incoloy 800HT (593 ± 5 MPa). In the AW condition, the fracture was observed in the region of the Incoloy 800HT HAZ, as shown in Table 5. After the PWHT, the fracture occurred in the region of the Incoloy 800 HT base metal with a tensile strength of 576 ± 5 MPa, which was still lower than the P91 and Incoloy 800 HT base metal. However, a fracture from the region of the Incoloy 800 HT base metal ensures that the joint is safe for USC boiler service after the PWHT. The percentage (%) elongation of the welded joint was also measured and found to be higher than the Incoloy 800 HT base metal for both AW (25 ± 2%) and PWHT (27 ± 3%) conditions. The yield strength of the welded joint was measured as 265 ± 4 MPa and 297 ± 5 MPa for the AW and PWHT joint, which was close to the yield strength value of the Incoloy 800HT base metal (285 ± 4 MPa). Base metals exhibited the highest tensile and yield strength, while the welded specimens have shown resembling results, which are reported in another study [48]. For a sub-size specimen, the fracture was observed in the center region of the WFZ, and the strength was measured as 565 ± 5 MPa and 549 ± 4 MPa for AW and PWHT conditions, respectively. The failure from the center region of the WFZ might be due to the presence of the solidification cracks, which showed the sudden propagation just after the yielding. Still, with the lower yield strength observed in AW and PWHT conditions, tensile strength was obtained in the reported range. This might suggest that the cracks were only dominant near the starting region of the weld bead due to the instability caused by the initiation of the keyhole and high heat input, or the liquid metal may have filled the cracks to slightly heal them, as represented in Figure 13a. It can be visualized from Figure 13b that there was backfilling as the major compositional elements in the area mapping analysis were evenly distributed in the region. Figure 14a–c depicts the crack along with the columnar dendritic microstructure, and the magnified view of the region shows the re-solidification in the crack Figure 14b–d [44]. The percentage of elongation measured for sub-size specimens were 198 ± 3 MPa and 206 ± 4 MPa for AW and PWHT, respectively, which was lower than the corresponding values of a standard tensile specimen. The percentage of elongation was also measured as lower than the standard tensile specimen, and was 22 ± 2% and 15 ± 2% for the AW and PWHT joint (Figure 12b).

**Figure 15:**
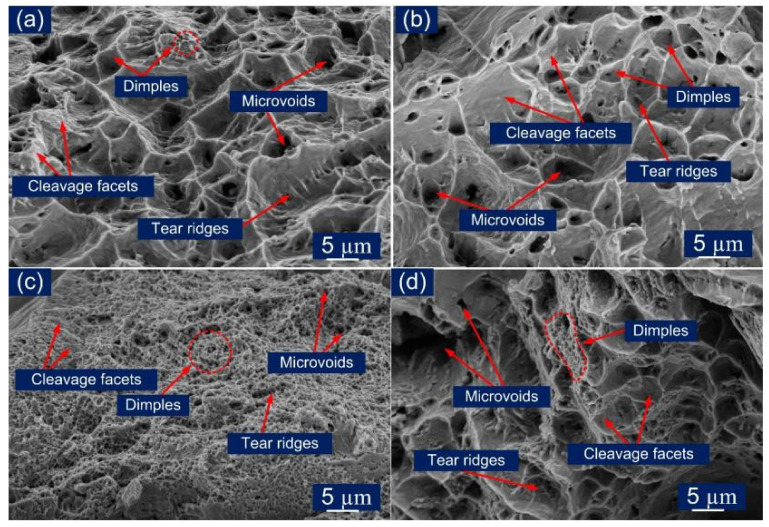
SEM fractography micrographs of (**a**) standard AW specimen, (**b**) standard PWHT specimen, (**c**) sub-size AW specimen, (**d**) sub-size PWHT specimen.

The fractured tensile specimen was also analyzed using the FE-SEM and is presented in Figure 15. The presence of the dimples at the fracture surface also supports the results (% elongation) that were obtained through the tensile tests. Figure 15a,b shows that failure from a ductile mode of fracture occurs in standard size specimens with Incoloy 800HT HAZ (AW) and the base metal (PWHT), as dimples were uniformly distributed. The fractured specimen in AW and PWHT conditions exhibits ductile dimples of varying size and depth, microvoids of varying size and depth, and tear ridges. The PWHT fractured specimen also shows the presence of a few brittle areas, i.e., cleavage facets (Figure 15b). While sub-size AW and PWHT specimens failing due to weld had experienced the intergranular and transgranular types of fracture, the presence of fewer dimples suggests slight ductile behavior (Figure 15c,d). The appearance of the fracture surface also supports the tensile test results. The fracture surface for the AW specimen exhibits a large area of the cleavage facets (Figure 15c), while after the PWHT, both fine dimples and microvoids were observed, along with the cleavage facets (Figure 15d).

#### 3.3.2. Microhardness Variation

**Figure 16:**
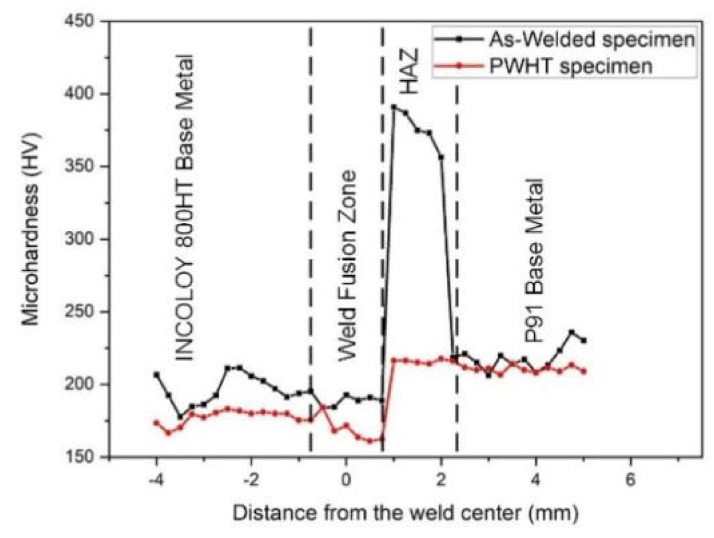
Microhardness variation obtained for AW and PWHT weldments.

The inhomogeneity in the microstructure obtained, due to the welding of dissimilar metals, impacts the mechanical property of the welds joint. Hence, microhardness analysis was conducted for the categorized welding zones. The comparison was done in the AW and PWHT states, as illustrated in Figure 16. The obtained microhardness for the base P91 and Incoloy 800HT was 245 ± 5 HV and 189 ± 5 HV, respectively. A comparatively lower hardness of 187 ± 3 HV was observed in the weld fusion zone of the AW joint, which was attributed to the austenitic microstructure and segregation of the alloying elements at the inter-dendritic boundaries. A uniform hardness variation was measured in the WFZ except for the Incoloy 800HT, which showed a slight increase in hardness that could be attributed to the formation of the unmixed zone due to differences in melting temperatures of parent base metals. As is apparent in the illustration, a notable variation in the measured hardness is evident along the weldment. The AW P91 side HAZ showed a significant increment in hardness with a peak hardness of 391 HV near the fusion boundary, i.e., CGHAZ. With the P91 CGHAZ being martensitic in nature, a higher hardness due to the presence of a slightly higher alloying concentration of Mo, W, C, and N was shown. The complete dissolution of MX and M_23_C_6_ precipitates in the region P91 CGHAZ formed under high temperature (>>A_c3_) increases the percentage of carbon and nitrogen in the solution matrix, hence causing the increase in hardness by solid solution strengthening. A similar observation has also been reported for P91 CGHAZ [36,49]. However, the hardness of P91 CGHAZ in the fusion welded joint was reported as much higher than the laser beam welded joint [29,50,51]. Gradually, a lower hardness in P91 FGHAZ was observed, varying from 387 HV to 356 HV with an average of 373 ± 12. The incomplete dissolution of the precipitates in FGHAZ as a result of the lower peak temperature (≥A_c3_) might be the cause of the poorer hardness in P91 FGHAZ than in the P91 CGHAZ. The minimum hardness in P91 HAZ was measured in ICHAZ, which experienced the temperature in between A_c1_ and A_c3_. The poor hardness of 218 HV in P91 ICHAZ was attributed to the re-tempering of martensite, grain and precipitate coarsening. In the AW condition, the carbide and nitride precipitates imparted the hardness to Incoloy 800HT, but being austenitic in nature, it exhibited a comparatively lower hardness than P91 and did not show any significant variation. Owing to this property of Incoloy 800HT, the HAZ hardness was measured as 195 HV, which is closely comparable to the Incoloy 800HT base metal hardness.

The tempering of the welded joint showed a significant effect on the hardness value of the P91 HAZ, while the hardness of the WFZ and Incoloy 800HT HAZ remained unaffected. After PWHT, the lowering in the hardness of P91 HAZ was significantly due to the tempering of martensite. The dissolved precipitates in the AW condition resurfaced while tempering. Hence, the CGHAZ of P91 after PWHT depicted a sharp decline of 174 HV in hardness compared to the AW state. A drastic reduction in the hardness of the P91 FGHAZ was also measured, with a hardness of 356 HV. However, the hardness of the P91 ICHAZ remains unaffected, and it was 218 HV. A similar trend of the hardness variation in P91 HAZ was also reported by Pandey et al. [52]. While theoretically, austenitic Incoloy 800HT and WFZ do not respond to PWHT, a slight reduction can be observed in the Incoloy 800HT side and even in the WFZ. This might be due to softening by carbide dissolution [44]. In addition, it might be argued that this reduction in hardness is due to ageing [45]. It can be concluded that PWHT is successful in reducing the hardness in the martensitic HAZ and reducing the hardness variation along the weldment.

#### 3.3.3. Charpy Impact Toughness

**Figure 17:**
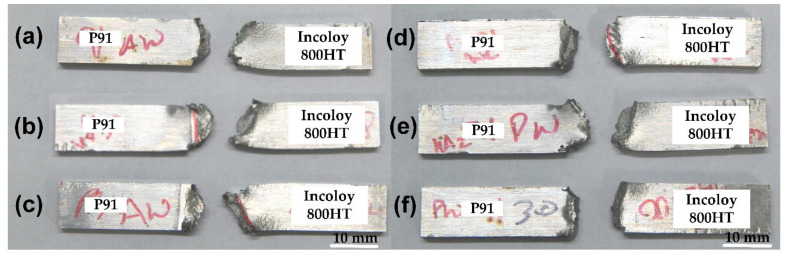
Samples fractured under Charpy impact testing from (**a**) WFZ (**b**) HAZ P91; (**c**) HAZ Incoloy 800HT; (**d**) PWHT WFZ; (**e**) PWHT HAZ P91; (**f**) PWHT Incoloy HAZ.

**Figure 18:**
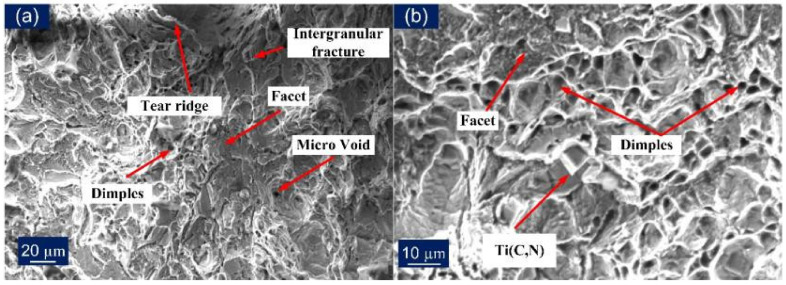
SEM fractography micrograph of specimens broken during Charpy impact toughness test (**a**) AW (impact at WFZ), (**b**) PWHT (impact at WFZ).

The energy absorbing capacity of the welded joint was evaluated by performing the Charpy impact toughness test at room temperature, and the results are shown in Table 6. The fractured impact tested specimen is shown in Figure 17. The impact toughness of the WFZ was measured as 65 ± 4 J in the AW condition, which was very low compared with the impact toughness of the P91 base metal (105 ± 4 J) and Incoloy 800 HT base metal (102 ± 5 J). The poor impact toughness value for the WFZ was attributed to the presence of solidification cracks along the SGBs and MGBs in the weld and segregation of the alloying elements, along with the inter-dendritic areas. However, the impact toughness of the WFZ obtained for the AW joint met the standard recommended value of 41 J (ASME standard) and >47 J (EN 1599:1997 standard) [53], which ensured that the impact toughness of the dissimilar laser welded joint of P91 and Incoloy 800 HT was qualified for boiler requirements. The fractured impact specimen showed the complete fracture of the specimen into two parts for both AW and PWHT joints (Figure 17). In dissimilar weldments, the results showed the maximum impact toughness for the P91 base metal compared to the WFZ and Incoloy 800 HT base metal. Meanwhile, the impact toughness of the P91 HAZ and Incoloy 800HT HAZ were measured as 102 ± 5 J and 75 ± 3 J, respectively (Table 6). The lower Incoloy 800HT HAZ impact toughness, in contrast with the P91 HAZ, was highly unexpected and suggested the formation of secondary carbide particles in the intergranular region due to the segregation of the alloying elements near the HAZ region [54]. The presence of precipitation in Incoloy 800HT HAZ hindered the dislocation movement, hence lowering the ductility and toughness [45]. In P91 HAZ, the impact toughness was measured as close to the P91 base metal. However, a dissimilar weld of P91 with austenitic grade steel with fusion-welded A-TIG and M-TIG process produced poor impact toughness, and it did not qualify the USC boiler requirements [50,55,56]. The PWHT showed an increase in the impact toughness of the P91 HAZ, and was measured at 108±6, while a drastic reduction was observed in the WFZ and Incoloy 800 HT HAZ. The impact toughness in the WFZ and Incoloy 800 HT HAZ were measured at 55 ± 2 J and 45 ± 3 J, respectively. The increase in impact toughness might be due to the tempering of the martensite. The increase in impact toughness of the WM after the PWHT was also reported in the previous study [56]. The impact toughness of the P91 HAZ was measured as higher than the P91 base metal. The much-reduced toughness value in Incoloy 800HT HAZ could be due to slight ageing, as the intergranular fracture mode was observed and is frequently due to the formation of secondary carbides in the matrix, dissolution of primary carbides, and phase transformation of TiC to Cr_23_C_6_ [23,54]. As illustrated in Figure 18a,b, the fractography analysis shows the formation of narrow and deep dimples, which confirms the ductile mode of failure mixed with the intragranular fracture. It seems complicated to comment on the loss of ductility. It could be due to either the presence of solidification cracks or due to the formation of secondary carbides.

## 4. Conclusions

The welding of dissimilar metals is a complicated task. The creep-strength-enhanced ferritic steels, such as P91, and nickel-based superalloys, such as Incoloy 800HT, have challenging weldability issues due to the difference in their physical and chemical properties. The application of such dissimilar welded joints motivates exploration into possible welding techniques and material combinations which may increase the power plant efficiency and reduce harmful emissions. The present work focused on dissimilar weld characterization using the laser beam welding process. The results concluded from tests conducted on the dissimilar welded joint of P91 and Incoloy 800HT can contribute to data collection for the behavior of dissimilar joining of martensitic steel and austenitic materials.

1. The significant finding was of solidification cracks occurring in the weld zone only, due to high heat input from laser welding and invariable cooling due to the presence of dissimilar alloys. The solidification temperature difference due to the keyhole effect can account for the cracking that was observed in the weld fusion zone. The segregation of elements, such as Ti, Si, and Nb, was observed in inter-dendritic regions, which caused cracking along solidification boundaries. In addition, the formation of columnar dendritic structures contributes to cracking.
2. Microstructural analysis of the weld fusion zone confirmed the presence of columnar, cellular, and equiaxed dendrites distributed in a random fashion. A substantial amount of detrimental elongated columnar dendritic structure was also witnessed. Formation of the unmixed zone near the P91 interface and partially mixed zone near the Incoloy 800HT interface were also observed. In addition, macrosegregation in the form of a peninsula and island was found at the weld fusion boundary of P91 and Incoloy 800HT.
3. A comparatively thicker transition zone was formed at the Incoloy 800HT weld fusion boundary. The heat-affected zone of P91 was clearly distinguishable into the CGAHZ, FGHAZ, and ICHAZ zones, while Incoloy 800HT had no distinguishable HAZ formation; however, thickening of grain boundaries was witnessed near the weld fusion boundary of Incoloy 800HT, due to the Ti related precipitation activity near the grain boundary. PWHT did not have any effect on the observed macrosegregation, UZ, TZ, and PMZ.
4. The tensile strength and impact toughness obtained for the laser beam welded dissimilar joint of P91, and Incoloy 800HT met the boiler requirement. Furthermore, the Incoloy 800HT base metal and HAZ were the parts of the welded joint with relatively weak tensile strength, while the welded joint with relatively poor impact toughness was the weld fusion zone.
5. The impact toughness of the weld fusion zone was obtained as 65 ± 4 J in the as-weld condition, which was lower than the parent metal P91 (105 ± 4 J) and Incoloy 800HT (102 ± 5 J). However, it was higher than the ASME standard value (>41 J) and EN 1599:1997 standard value (>47 J), which is required for the safe operation of power plants. The weld fusion zone showed poor impact toughness as compared with other zones of the weldments, and the inferior impact toughness of the weld fusion zone might be due to cracks in the WFZ and secondary carbide formation. The average impact toughness of the P91 HAZ (102 ± 5 J) and Incoloy 800HT HAZ (75 ± 3 J) was measured as lower than the respective base metal impact toughness. The weld fusion zone and Incoloy 800HT HAZ exhibited a noteworthy decrease in impact toughness after the PWHT; however, the impact toughness of P91 HAZ showed a minute increase.
6. For a standard specimen, the tensile strength was obtained 546 ± 6 MPa and 576 ± 5 MPa for the as-welded and PWHT joints, respectively, which was lower than the base metals P91 and Incoloy 800HT. The standard tensile specimen of both the AW and PWHT condition failed from the Incoloy 800HT side, establishing it as the weakest part of the weld. For the subsize sample, tensile strength was measured 565 ± 5 MPa and 549 ± 4 MPa for as-weld and PWHT joints. The lowest tensile strength of 549 ± 4 MPa was observed for the PWHT subsize specimen owing to the presence of solidification cracks in the weld center as sudden fracture occurred. The cracks present in the weld center made it difficult to establish the effect of PWHT on the tensile strength.
7. Microhardness characterization confirmed the presence of different zones formed as per the different ranges of temperature exposure while welding, which led to the hardness variation across the welded joint for both as-welded and PWHT joints. In the AW condition, a peak hardness of 391 HV was observed in P91 CGHAZ nearest to the fusion boundary owing to the complete dissolution of precipitates at high welding temperature. The minimum hardness of 218 HV was observed in ICHAZ due to the grain and precipitate coarsening. The Incoloy 800HT in AW condition had comparatively low hardness, and its HAZ had the hardness of 195 HV, which was comparable to the base metal owing to the presence of the austenitic structure. The weld fusion zone also had lower hardness in the uniform range of 187 ± 3 HV, again due to the austenitic structure of the weld fusion zone. The PWHT observed a minute effect on the hardness value of the weld fusion zone and Incoloy 800HT HAZ; however, a drastic reduction in P91 CGHAZ/FGHAZ was obtained.
8. The outcome of this work can be worthy of exploring the laser-welded joining of the P91 and Incoloy 800HT as complete penetration was obtained in a single pass which is difficult to get using any conventional welding technique. In addition, the welding preparation is simple and avoids the use of any filler material. The focused laser beam single pass gives a narrower weld bead and narrower HAZ, limiting the mechanical properties’ variation.

## Figures and Tables

**Table 1:** Chemical composition of as-received P91 and Incoloy 800HT (wt.%).

Materials	Fe	C	Si	Mn	Cr	Mo	Ni	Al	Nb	Ti	V	W
P91	88.8	0.0997	0.160	0.389	8.40	0.991	0.276	0.0111	0.0687	0.0023	0.207	0.0179
Incoloy 800HT	43.4	0.0838	0.464	1.02	21.1	0.124	32.7	0.353	0.0303	0.494	0.0501	0.0325

**Table 2:** Laser welding parameters.

Parameters	Values
Power	4000 W
Speed	7 mm/s
Heat input	571 J/mm
Beam spot diameter	0.6 mm
Focal length	150 mm
Weld penetration	6 mm
Weld angle	90°

**Table 3:** Mechanical properties of the as-received base material.

Specimens	Tensile Strength (MPa)	Yield Strength [offset 0.2%] (MPa)	Elongation (%)	Reduction of Area (%)	Impact Toughness (J)
P91 steel base metal	687 ± 8	604 ± 6	39 ± 2	79 ± 3	105 ± 4
Incoloy 800HT base metal	593 ± 5	285 ± 4	10 ± 3	65 ± 3	102 ± 5

**Table 4:** Tensile test results.

Specimens	Tensile Strength (MPa)	Yield Strength [offset 0.2%] (MPa)	Elongation (%)	Reduction in Area (%)
AW (Standard specimen)	546 ± 6	265 ± 4	25 ± 2	48 ± 4
AW (Subsize specimen)	565 ± 5	198 ± 3	22 ± 2	48 ± 5
PWHT (Standard specimen)	576 ± 5	297 ± 5	27 ± 3	53 ± 3
PWHT (Subsize specimen)	549 ± 4	206 ± 4	15 ± 2	40 ± 4

**Table 5:** Fracture locations for the tensile tested specimen in different conditions.

Specimen	AW	PWHT
Standard	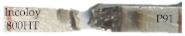 From Incoloy 800HT HAZ	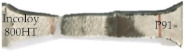 From Base Metal
Sub-size	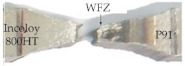 From WFZ	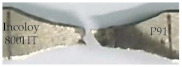 From WFZ

**Table 6:** Impact toughness obtained in Charpy impact testing.

Specimens	Impact Zone	Charpy Impact Toughness (J)
**AW**	WFZ	65 ± 4
	P91 HAZ	102 ± 5
	Incoloy 800HT HAZ	75 ± 3
**PWHT**	WFZ	55 ± 2
	P91 HAZ	108 ± 6
	Incoloy 800HT HAZ	45 ± 3

## Data Availability

Not applicable.
